# A Peptide Derived from Phage Display Library Exhibits Antibacterial Activity against *E. coli* and *Pseudomonas aeruginosa*

**DOI:** 10.1371/journal.pone.0056081

**Published:** 2013-02-11

**Authors:** Shilpakala Sainath Rao, Ketha V. K. Mohan, Chintamani D. Atreya

**Affiliations:** Section of Cell Biology, Laboratory of Cellular Hematology, Division of Hematology, Center for Biologics Evaluation and Research, US Food and Drug Administration, Bethesda, Maryland, United States of America; The Scripps Research Institute and Sorrento Therapeutics, Inc., United States of America

## Abstract

Emergence of drug resistant strains to currently available antibiotics has resulted in the quest for novel antimicrobial agents. Antimicrobial peptides (AMPs) are receiving attention as alternatives to antibiotics. In this study, we used phage-display random peptide library to identify peptides binding to the cell surface of *E. coli*. The peptide with sequence RLLFRKIRRLKR (EC5) bound to the cell surface of *E. coli* and exhibited certain features common to AMPs and was rich in Arginine and Lysine residues. Antimicrobial activity of the peptide was tested *in vitro* by growth inhibition assays and the bacterial membrane permeabilization assay. The peptide was highly active against Gram-negative organisms and showed significant bactericidal activity against *E. coli* and *P. aeruginosa* resulting in a reduction of 5 log_10_ CFU/ml. In homologous plasma and platelets, incubation of EC5 with the bacteria resulted in significant reduction of *E. coli* and *P. aeruginosa*, compared to the peptide-free controls. The peptide was non-hemolytic and non-cytotoxic when tested on eukaryotic cells in culture. EC5 was able to permeabilize the outer membrane of *E. coli* and *P. aeruginosa* causing rapid depolarization of cytoplasmic membrane resulting in killing of the cells at 5 minutes of exposure. The secondary structure of the peptide showed a α-helical conformation in the presence of aqueous environment. The bacterial lipid interaction with the peptide was also investigated using Molecular Dynamic Simulations. Thus this study demonstrates that peptides identified to bind to bacterial cell surface through phage-display screening may additionally aid in identifying and developing novel antimicrobial peptides.

## Introduction

Drug resistance among pathogenic bacteria is on the rise and antibiotics to combat these microbes are becoming limited. New antibacterial agents with novel mechanisms of action and biological targets need to be developed. AMPs are a new class of antimicrobial agents that has stimulated the interest of many investigators as a substitute for traditional antibiotics [1]. Most of the AMPs are components of innate immunity evolved millions of years ago as primary defense to combat microbial challenge [2]. Though human defensins and cathelicidins from higher vertebrates are the most studied AMPs, significant number of diverse AMPs from plants, vertebrates, and invertebrates are also receiving lots of attention [3], [4]. AMPs have a positive net charge and interact with the negatively charged membranes, leading to destabilization and permeabilization of the cell membrane [5]–[7].

Though the exact mode of action of the AMPs has not been fully understood, several models for the interaction of AMPs with membranes such as the “carpet model”, “toroid pore” model and the “barrel-stave” model have been proposed [8]. The mechanism of action of AMPs against Gram-negative bacteria has been extensively studied [9]. Many of the antimicrobial peptides act by binding to the negatively charged lipopolysaccharide (LPS), thereby rendering the bacterial membrane permeable. Additional peptide molecules present outside the membrane gains entry into the cell and integrate into the cytoplasmic membrane resulting in cell death [10].However, some AMPs may act differently under different conditions [1].

Recently, scientists have focused their research on screening novel AMPs by using combinatorial libraries and computational approaches for antimicrobial drug discovery and design [11]–[17]. Phage-display serves as a valuable tool for the selection of peptides binding to surface epitopes on whole cells [18]–[21]. This approach could allow the selection of bacterial membrane ligands with antimicrobial activity from a large peptide library [22]. Short synthetic peptides are gaining importance as probes for detection and therapeutic purposes and thereby offer excellent templates for future drug design. In this study we used a 12-mer phage-display library to identify peptides binding to the whole cell surface of *E. coli*. We identified a novel peptide that showed binding to *E. coli* cells. The peptide exhibited antimicrobial activity against Gram-negative organisms and showed significant bactericidal activity against *E. coli* and *P. aeruginosa*. Furthermore the peptide was tested for membrane permeabilization, specificity and toxicity against mammalian cells.

## Materials and Methods

### Bacterial culture and reagent

All bacterial strains used in this study originated from ATCC (Manassas, Virginia): *S. aureus* (ATCC 25923; ATCC 35548), *S. epidermidis* (ATCC 35983), *E.coli* (ATCC 700928; ATCC 25922), *P. aeruginosa* (ATCC 27853; ATCC 12121), *K. pneumoniae* (ATCC 10031; ATCC 13885) and *B. cereus* (ATCC 11778). Cultures were maintained and subcultured periodically on nutrient agar plates and stored at 4 to 8°C until tested. Stock cultures of all bacteria were stored in tryptic soy broth with 10% glycerol at −70°C. Log-phase cultures of bacteria grown in Miller's Luria-Bertani (LB) broth (Mediatech Inc, Herndon, VA) were concentrated by centrifugation at 3000×g and dilutions were prepared with phosphate-buffered saline (PBS; pH 7.4) (Mediatech Inc, Herndon, VA). In all experiments bacterial titers were estimated by optical density and confirmed by quantitative culture by plating on nutrient agar plates.

### Biopanning of phages binding to *E. coli*

Phage library displaying 12-mer random peptides fused to pIII coat protein (New England Biolabs Ipswich, MA) was used for this study. Biopanning and amplification of the phages was performed as described earlier with some modifications [23]. In brief, the phage library was depleted of clones binding to *S. aureus* ATCC 25923. For the first round of panning, 96-well plates were coated with whole cells of *E. coli* ATCC 700928 resuspended in PBS pH 7.4 and exposed to the phage library diluted in TBST at a final concentration of 2×10^10^ (100 µl/well). Unbound phages were removed by washing 10 times with TBST. Subsequently bound phages were eluted by adding 100 µl elution buffer (100 mM HCl) for 5 min at room temperature. The eluate containing the bound phage neutralized with 1 M Tris (pH 8.0) was collected. The titer of the phage was determined by plating them on LB X-gal/IPTG plates and the phages were amplified in *E. coli* ER2738 and purified with polyethylene-glycol precipitation. In each round of panning, the titer of the phages in washing buffer and that in the elution buffer was determined, and their ratio was analyzed to evaluate the enrichment efficiency.

### DNA sequencing and peptide synthesis

Phages from sixth round of biopanning were used for preparing phage stocks to isolate phage genomic DNA for nucleotide sequencing. The DNA sequences were translated into amino acids by using ‘Gene Runner’ software (www.generunner.net). Phage peptides were aligned using CLUSTAL W multiple sequence alignment program (http://www.ebi.ac.uk/Tools/msa/clustalw2/) [24], [25]. Peptides were synthesized at our Core Facility in CBER, FDA, biotinylated with a C6-linker and purified by high-pressure liquid chromatography (HPLC).

### Assays for detection of peptide binding to *E. coli* cells

ELISA-Log-phase cultures of bacteria were centrifuged at 3,000×g. The cell pellet was resuspended and 10-fold serial dilutions were made in 1× PBS. Bacterial cell suspension at a concentration of 10^3^ CFU was coated to the wells of 96-well micro plate (Becton Dickinson, Bedford, MA) and incubated overnight at RT. Subsequently, cells were fixed in ethanol (Aldrich Chemical Company, Milwaukee, WI) for 10 min and the plates were air-dried. Wells were blocked with 5% BSA (Sigma, St. Louis, MO) for 60 min at RT, rinsed with PBS and then EC5 peptide suspended in PBS at a final concentration of 50 µg/ml was added to all the wells and incubated for 15 min. Following incubation, wells were washed 3 times with PBST buffer (PBS pH 7.4, 0.01% Tween 20). The wells were further incubated with 1∶10,000 dilution of streptavidin-HRP conjugate (Upstate, Temecula, CA) for 15 min and washed with PBST buffer. Tetramethylbenzidine (TMB) membrane peroxidase substrate (Zymed Laboratories, Carlsbad, CA) system was used to detect the enzyme label in accordance with the manufacturer's instructions. The color development in the 96-well plate was recorded by using Synergy 4™ BioTek micro plate reader (BioTek Instruments, Winooski, VT) at 490 nm wavelength.

*Fluorometry using Qdot nanocrystals-*The assay was carried out as described earlier [23], [26]. Bacterial cell suspensions at a concentration of 10^3^ CFU were incubated with EC5 (50 µg/ml) in an eppendorf tube at room temperature for 60 min. Following three washes with 1×PBS, pH 7.4 the pellet was resuspended in 100 µl of 1∶10,000 dilution of streptavidin-conjugated Q dots (QD 605) solution (Invitrogen, Gaithersburg, MD). The fluorometric counts were measured using Synergy 4™ BioTek micro plate reader (BioTek Instruments, Winooski, VT).

### Bioinformatic tools for peptide characterization

Physicochemical properties of the peptide (Molecular weight and pI) were predicted using the Compute pI/Mw tool expasy server (http://web.expasy.org/compute_pi/). Hydrophobicity and net charge of the peptides were predicted using Antimicrobial peptide database server (http://aps.unmc.edu/AP/prediction/prediction_main.php) [17]. Homology modelling was done using the (PS) 2v2: Protein Structure Prediction Server [27]. (PS) 2 is an automated server that builds 3D models using the package MODELLER.

### Evaluation of bactericidal activity of the EC5 peptide

*In vitro* antibacterial activity of EC5 peptide. The test microorganisms mentioned earlier in the experimental procedures were used to evaluate the bactericidal activity of EC5 peptide. The minimum inhibitory concentrations (MICs) of the peptide was determined by standard dilution assay as recommended by Clinical and Laboratory Standard Institutes (CLSI) guidelines [28]. Overnight cultures of bacterial cells were diluted in cation-supplemented Mueller-Hinton broth (MHB) (Becton Dickinson, Sparks, MD) and grown to mid-logarithmic phase on an orbital shaker (37°C) and diluted to a 0.5 McFarland standard to a final volume of 1 ml. Decreasing concentrations of the peptide were incubated with the microorganisms. Results were recorded by visual inspection after 24 h of incubation at 37°C. Assay was repeated three separate times to ensure reproducibility. Since EC5 exhibited antibacterial activity against *E. coli* and *P. aeruginosa*, time-kill kinetics of the *E. coli* and *P. aeruginosa* strains were examined. EC5 concentrations 0x, 0.5x, 1x, and 2x MIC were incubated with logarithmic phase of bacteria of approximately 10^5^ CFU/ml in an orbital shaker for 48 h at 37°C. Samples were drawn and plated on NA plates. Polymyxin B was used as a positive control and peptide with no antibacterial activity (PD-1) [29] from our previous studies was used as a negative control. Additional MIC determinations were performed using cation-supplemented Mueller-Hinton broth.

### Antibacterial activity in blood matrices

The antibacterial activity of the peptide in the presence of homologous plasma and platelets were assessed. Platelet Concentrates (PCs) in bags were obtained from the National Institutes of Health (NIH) Blood Bank, Department of Transfusion Medicine, Clinical Center (NIH, Bethesda, MD) and stored at room temperature as described earlier [29]. Plasma was isolated from the PC bag by collecting 25 ml of PCs and subjecting the sample to a low speed centrifugation step to separate PLT-rich plasma from plasma. For all assays log-phase cultures of bacteria grown in Luria-Bertani (LB) broth were centrifuged at 3000×g and suspended in 1×PBS pH 7.4. Bacterial titers were estimated by measuring the optical density (OD) and microscopy. Approximately 10^5^ CFU/ml of each bacterial strain was spiked into 0.1 ml of plasma or platelets and incubated with peptide at concentrations ranging from 0 to 50 µg/ml. Incubation was carried out at room temperature for 2 h on a shaker. At the end of 2-h exposure period, a fixed volume of the suspension was plated on NA plates, and incubated at 37°C for 18 to 24 h. Bactericidal activity was measured by log-reduction of viable bacteria.

### Measurement of Hemolytic activity

The ability of EC5 to induce hemolysis of chicken red blood cells (ch-RBCs) was assessed as previously described [30]. Ch-Red blood cells were purchased from Lampire Biological Labs Inc, Pipersville, PA and the ch-RBCs were harvested under the manufacturer's established cGMPs. The purchased ch-RBCs were washed with phosphate-buffered saline (PBS) and a 1% (vol/vol) suspension was made with PBS. 100 µl of this RBC suspension were transferred to 96-well microtiter plates. Two-fold serial dilutions of the peptide samples (range: 25 to 500 µg/ml) were added to the RBCs and the reaction mixture was incubated at 37°C for 24 h in microtiter plates. Optical density was measured with a microtiter plate reader (BioTek Instruments, Winooski, VT) to monitor RBC lysis. Cells incubated with PBS alone served as control and cells that were lysed using 0.1% Triton X-100 was used as positive control.

### Toxicity of peptide for Eukaryotic cell

Toxicity of EC5 towards MDCK (Madin Darby Canine Kidney ATCC CCL-34) cells and Vero cells (ATCC CCl-81) was tested by PrestoBlue™ Cell Viability assay (Invitrogen, Carlsbad, CA) according to the manufacturer's protocol. Briefly MDCK and Vero cells were cultured in Eagle's Minimum Essential Medium (EMEM) (Invitrogen, Carlsbad, CA) and Dulbecco's modification of Eagle medium respectively. The medium was supplemented with 10% fetal bovine serum, 2 mM glutamine, 100 units/ml of penicillin, and 100 units/ml streptomycin was plated in wells. Peptides were added at various concentrations, ranging from 0 to 500 µg/ml, and incubated overnight with the cells at 37°C in a 5% CO_2_ atmosphere. Ten percent DMSO was used as a positive control and untreated cells served as negative control. PrestoBlue™ Cell Viability reagent was added and the fluorescence read using Synergy 4™ BioTek micro plate reader (BioTek Instruments, Winooski, VT).

### Outer membrane permeabilization assay

Membrane-permeabilizing activity of the peptide was determined using the fluorescent dye N-phenyl-1-napthylamine (NPN) assay, as described earlier [31], with intact cells of *E. coli* and *P. aeruginosa*. The increase in fluorescence due to partitioning of NPN uptake into outer membrane (OM) was measured by addition of increasing concentrations of peptide. Polymyxin B (PMB) was taken as a positive control due to its outer membrane permeabilising properties. All experiments were performed three times. *S. aureus* was used as a negative control.

### Membrane permeability as assessed by propidium iodide (PI) and SYTO9 uptake-based assay

The effects of EC5 on the membrane integrity of *E. coli* and *P. aeruginosa* cells were assessed using the LIVE/DEAD *Bac*light™ kit (Invitrogen, Oregon) according to the manufacturer's protocol. Membrane integrity of the cells after the addition of different concentrations of peptide EC5 was measured using fluorescence micro plate reader (BioTek Instruments, Winooski, VT). Experiments were done in triplicates. PMB was used as a positive control.

### Membrane depolarization assay (Δψ)

Membrane depolarization activity (Δψ) of the peptide was determined with intact *E. coli* and *P. aeruginosa* cells and the membrane potential-sensitive fluorescent dye, 3,3′-dipropylthiadicarbocyanine Iodide (diSC (3)5) (Invitrogen, Oregon), according to the method described by Sims et al [32]. Briefly, bacterial cells of mid-logarithmic phase were harvested by centrifugation and washed twice with HEPES buffer (5 mM HEPES, 5 mM glucose, pH 7.6). The cells were suspended in the same buffer to an OD of 0.05. The cell suspension was incubated with 0.4 mM diSC (3)5 until maximal uptake of the dye. 100 mM KCl was added to equilibrate the cytoplasmic and external potassium ion concentration of peptide, and fluorescence was monitored at an excitation wavelength of 622 nm and an emission wavelength of 670 nm. Polymyxin B was used as a positive control and *S. aureus* cells were used as negative control. Simultaneously the cells were plated on NA plates and incubated at 37°C overnight to assess the number of CFU.

### Determination of ATP inhibition

The effects of EC5 on ATP inhibition as a measure of metabolic activity of *E. coli* and *P. aeruginosa* cells was determined by using BacTiter-Glo assay kit™ (Promega) according to the manufacturer's protocol. The luminescent signal correlates with the number of viable microbial cells based on the amount of ATP present after the addition of different concentrations of the peptide. Polymyxin B was included as a positive control.

### Molecular Dynamic simulations

*In-silico* docking studies of EC5 with lipid bilayers were conducted using Cluspro 2.0 version software [33] and Hex protein docking [34]. Files for POPE (1-palmitoyl-2-oleoyl-phosphoethanolamine) and POPG (1-palmitoyl-2-oleoyl-phosphatidylglycerol) were accessed from Tieleman website (http://moose.bio.ucalgary.ca) as described [35]. Structural files for EC5 were downloaded in PDB file format from (PS) 2v2: Protein Structure Prediction Server. Models of interaction of EC5 with lipid bilayers were generated by the Hex docking server (http://hexserver.loria.fr/) and Cluspro protein-protein docking server (Version 2.0). The rigid body docking is performed, using ZDOCK based on the fast Fourier transform correlation techniques. The scoring function of ZDOCK is based on shape complementarities, electrostatic potentials and desolvation terms. Filtering is performed using pair wise root mean square deviation clustering and empirical free energy functions. The ligand is minimized by the CHARMM algorithm in the presence of receptor. The 3D Model structures were visualized using PYMOL (version 0.99; http://www.pymol.org).

### Statistical analyses

Assays described here were performed at least three independent times. Mean values ± SD (Standard Deviation) was calculated using GraphPad prism 5. A two-way ANOVA was used to determine significance. Values were considered significant when p<0.05.

## Results

### Phage-display selection of peptides binding to *E. coli*

We used a 12-mer random phage display library to affinity select for peptides binding to the cell surface of *E. coli*. In order to eliminate non-specific binding phages and to select peptides that bind specifically to *E. coli*, we used a subtractive phage-display approach where the library was first pre-adsorbed against *S. aureus* ATCC 25923 to eliminate phages binding to the Gram-positive cell surface. The remaining phage library was then used to affinity select for peptides binding to whole cell surface of *E. coli* ATCC 700928. Six rounds of biopanning were performed and the enrichment level was determined prior to amplification by *E. coli* ER2738 infection. Enrichment level was monitored after each round by determining the titer of eluted phages. There was an increase in recovery rate after each round of selection indicating effective enrichment of the phage clones. After six rounds of biopanning, individual phage clones were isolated from which genomic DNA was extracted and sequenced. Five of the clones encoded the same peptide sequence RLLFRKIRRLKR, hereafter referred as EC5, and the remaining clones each encoded unique peptide sequences (Table 1). The amino acid sequences of the clones were aligned using ClustalW.

**Table 1:** Characteristics of various peptides identified by phage-display.

Clone	Sequence	Frequency	Net charge	Hydrophobicity	MW*
EC2	SGHQLLLNKMPN	1/10	+2	33%	1345.59
EC5	RLLFRKIRRLKR	5/10	+7	41%	1994.58
EC6	MDMRTTDIRDTS	1/10	−1	25%	1441.61
EC8	RNHPATLTGTGG	1/10	+2	16%	1175.28
EC9	GILSELGKALGG	1/10	0	41%	1174.36
EC10	GAPALSTPPLSR	1/10	+1	33%	1148.35

* MW – Molecular weight (theoretical).

### EC5 binds to *E. coli* and *P. aeruginosa*

The binding ability and specificity of EC5 peptide was assessed against a panel of bacteria using ELISA and Fluorometry using Q-dots.

**Figure 1:**
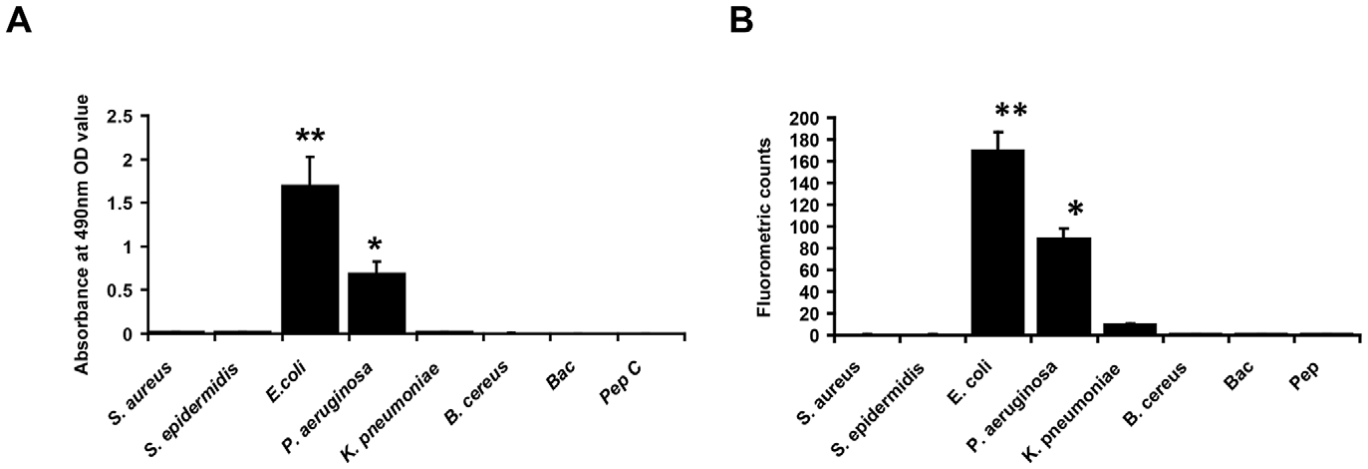
Binding efficiency of EC5 to different bacteria. A. ELISA based assay– 96 well microtiter plates were coated with six bacteria and incubated with the biotinylated peptide and the binding was detected using strepatavidin–HRP and developed using TMB substrate. (**–p<0.001, *–p<0.05). B. Fluorometry based assay– binding of EC5 to different bacteria was detected using streptavidin-conjugated Q dots. Results are presented as Mean±SD. (** – p<0.0001, *– p<0.001). Bac – Bacteria without peptide, Pep – Peptide without bacteria were used as controls.

*ELISA-based binding analysis of EC5-*Binding affinities and specificity of the synthesized peptide was analyzed by whole-cell ELISA. Peptide showed significant binding efficiency to *E. coli* cells (p<0.001) as seen in the Fig. 1A. Interestingly the peptide also showed binding to *P. aeruginosa* cells (p<0.05). However, EC5 did not bind to the Gram-positive *S. aureus*, *S. epidermidis*, *B. cereus* and to the Gram-negative *K. pneumoniae*.

*Qdot-based fluorometry as a confirmatory assay for the binding of EC5 to E. coli-*Binding of the peptide to the bacteria was detected using Qdot-nanocrystal cores conjugated to streptavidin. Analysis of the bacterial suspension binding to the peptide was performed in a micro-well plate using a fluorescence plate reader (Synergy 4™, Biotek, USA). Excitation was set to a spectral range of 360–485 nm and emission was at 605 nm. Fluorometric analysis revealed that EC5 was able to bind to *E. coli* as indicated by the significantly higher level of fluorometric counts (p<0.0001) (Fig. 1B). The analysis also confirmed that EC5 binds to *P. aeruginosa* cells.

### Peptide EC5 shows features of antimicrobial peptide

**Figure 2:**
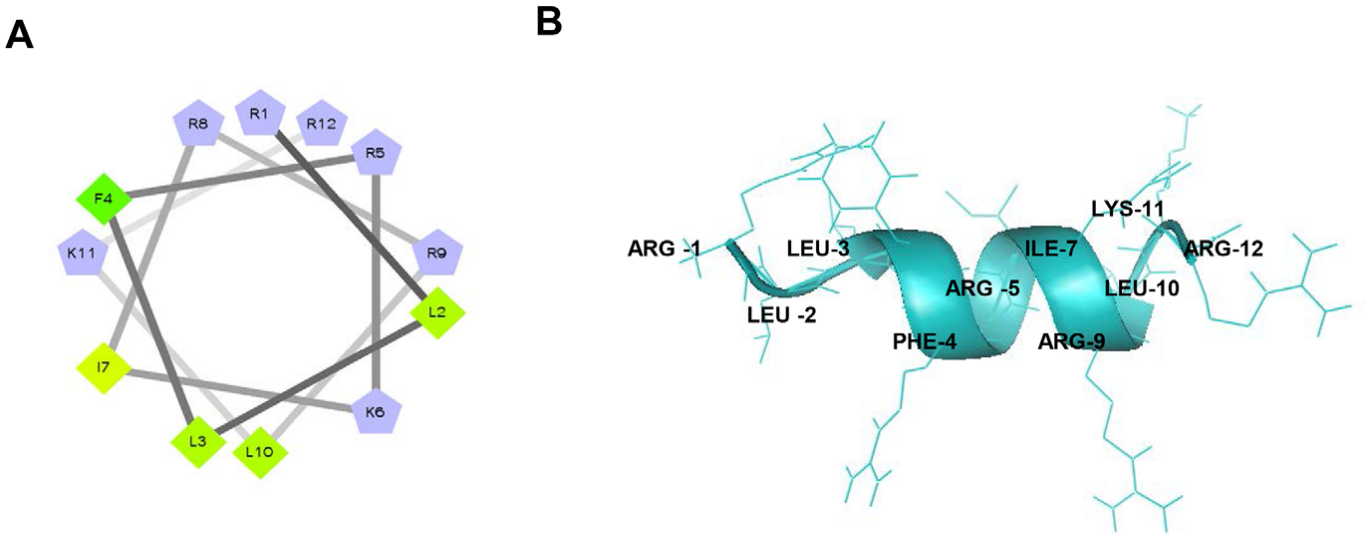
Structure and properties of EC5. A. Edmundson helical wheel presentation of 12-mer EC5. Hydrophobic residues are represented by diamonds and positive charge as pentagons. The most hydrophobic residue is green, with the amount of green decreasing proportionally to the hydrophobicity with least hydrophobic being yellow. B. Ribbon and surface representation of EC5. Ribbon model shows α-helical peptide as the conformation. Green-most hydrophobic. Secondary structure of the peptide was determined and viewed using PYMOLv0.99.

The peptide EC5 exhibited some properties of antimicrobial peptides: cationic with a net positive charge (+7) and a total hydrophobic ratio of 41% (http://aps.unmc.edu/AP/prediction/prediction_main.php) (Table 1) [17]. By using feature selection method and sequence alignment as a method for prediction of antimicrobial peptide, it was found that EC5 may exhibit antimicrobial properties (http://amp.biosino.org/) [36]. The peptide showed no significant similarity to other sequences in the Antimicrobial Peptide Database. Secondary structure of EC5 was determined and shown to have α-helix conformation (Fig. 2) with a molecular formula of C_75_H_139_N_29_O_13_. Helical wheel presentation of the peptide was illustrated using the program: (http://rzlab.ucr.edu/scripts/wheel/wheel.cgi).

### Antimicrobial activity of EC5

**Figure 3:**
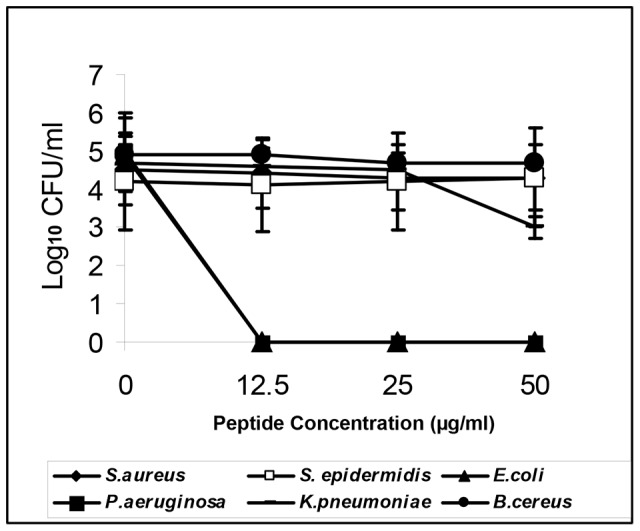
Effects of EC5 on the growth of different bacteria. Different concentrations of peptide EC5 was added to log phase cultures of bacteria and their growth monitored after 2 h. Numbers indicate reduction in log_10_ CFU/ml.

As EC5 demonstrated features common to an antimicrobial peptide we tried to investigate whether EC5 could exert antimicrobial effect *in vitro*. Mid-logarithmic phase cultures of bacteria with an inoculum of 10^5^ CFU/ml were incubated with peptide concentrations ranging from 0 to 50 µg/ml in a shaker incubator for 2 h. Subsequently the samples were plated on NA agar plates and CFU were determined. EC5 showed a reduction of 5 log_10_ CFU/ml of *E. coli* and *P. aeruginosa* at peptide concentrations of 12.5, 25 and 50 µg/ml as observed by absence of colonies on NA plates (Fig. 3). However it showed no activity against any of the other bacteria tested even at 50 µg/ml.

MIC of EC5 was determined for the reference strains (Table 2). EC5 was highly active against *E. coli* ATCC 700928 and ATCC 25922 with a MIC of 8 µg/ml. *P. aeruginosa* ATCC 27853 growth was inhibited at a MIC of 8 µg/ml and *P. aeruginosa* ATCC 12121 with a MIC range of 16–32 µg/ml. MIC of EC5 against *K. pneumoniae* was shown to be at 64–128 µg/ml. Minimum Bactericidal Concentration (MBC) of EC5 tested against *E. coli* ATCC 700928, ATCC 25922 and *P. aeruginosa* ATCC 27853 was 8 µg/ml indicating that EC5 is a potent bactericidal agent. However it was not active against *S. aureus*, *S. epidermidis*, and *B. cereus*.

**Table 2:** MIC of EC5 against bacteria (µg/ml).

Organism	MIC
*S. aureus* ATCC 25923	>128–256
*S. aureus* ATCC 35548	>128–256
*S. epidermidis* ATCC 35983	64
*B. cereus* ATCC 11778	64
*E. coli* ATCC 700928	8
*E. coli* ATCC 25922	8
*P. aeruginosa* ATCC 27853	8
*P. aeruginosa* ATCC 12121	8–16
*K. pneumoniae* ATCC 10031	32–64
*K. pneumoniae* ATCC 13885	32–64

**Figure 4:**
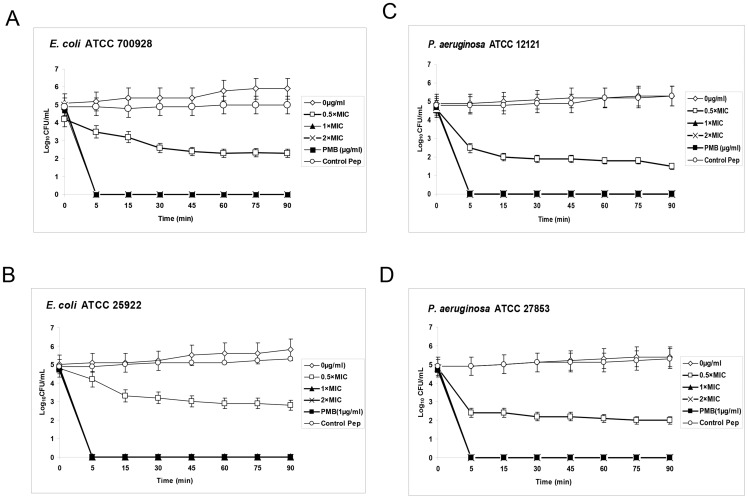
Killing kinetics of EC5 at different concentrations. A. *E. coli* ATCC 700928, B. *E. coli* ATCC 25922, C. *P. aeruginosa*, ATCC 12121, D. *P. aeruginosa* ATCC 27853. The curve represents surviving cell concentrations plotted against time.

Time-kill kinetic studies of EC5 was comparable to polymyxin B and exhibited most rapid bactericidal activity against *E. coli* ATCC (700928; 25922) and *P. aeruginosa* ATCC (12121; 27853) (Fig. 4) with complete inhibition after 5 min incubation with the peptide and showed no regrowth until 24 hrs. EC5 showed bactericidal activity in a dose-dependent manner. MBC concentration of 8 µg/ml showed complete killing of *E. coli* while growth inhibitory concentration of 4 µg/ml showed reduction of 2–3 log_10_ CFU/ml at around 45 min of exposure to the peptide. Control peptide with no antimicrobial activity was used as negative control.

### EC5 demonstrates bactericidal activity in the presence of plasma and platelets

**Figure 5:**
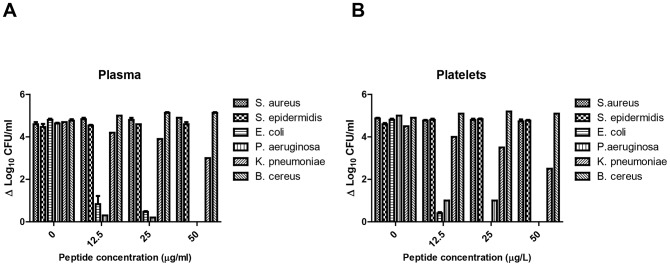
Effect of EC5 on different bacteria. A. Plasma, B. Platelets. Blood matrices were spiked with bacteria and incubated with different concentrations of EC5. Growth was monitored after 2 h by plating them onto NA plates.

Peptides that exhibit antimicrobial activity in conventional media may lose its activity in biological media. Hence we tested EC5 for its bactericidal activity in platelet and plasma samples spiked with the test bacteria as mentioned in materials and methods. The results illustrated in Fig. 5A suggest that EC5 was able to bring around 5 log_10_ CFU/ml reduction of *E. coli* and *P. aeruginosa* in plasma at 50 µg/ml. *E. coli* showed 4.5 and 3.5 log_10_ reduction at peptide concentrations of 25 and 12.5 µg/ml and *P. aeruginosa* showed around 4 and 3-log_10_ CFU/ml reduction at 25 and 12.5 µg/ml respectively. *K. pneumoniae* showed approximately a 2.5-log_10_ reduction in plasma when EC5 was at 50 µg/ml and around 1.5-log_10_ reduction at 25 µg/ml. However EC5 failed to cause a significant reduction of *S. aureus*, *S. epidermidis* and *B. cereus* even at 50 µg/ml.

EC5 at 50 and 25 µg/ml concentrations in platelets completely inhibited *E. coli* and showed no detectable colonies, while 12.5 µg/ml caused a reduction of 4.5 log_10_ CFU/ml compared to controls (Fig. 5B). The dose-dependent efficacy of EC5 against *P. aeruginosa* spiked in platelets was also evident with reduction of 4 log_10_ CFU/ml at peptide concentrations of 25 and 12.5 µg/ml. *K. pneumoniae* showed reduction of approximately 2.5, 1.5 and 1 log_10_CFU/ml at EC5 of 50, 25, 12.5 µg/ml, respectively. No significant reduction was seen with *S. aureus*, *S. epidermidis* and *B. cereus* even at high concentrations.

### Hemolytic activity

**Figure 6:**
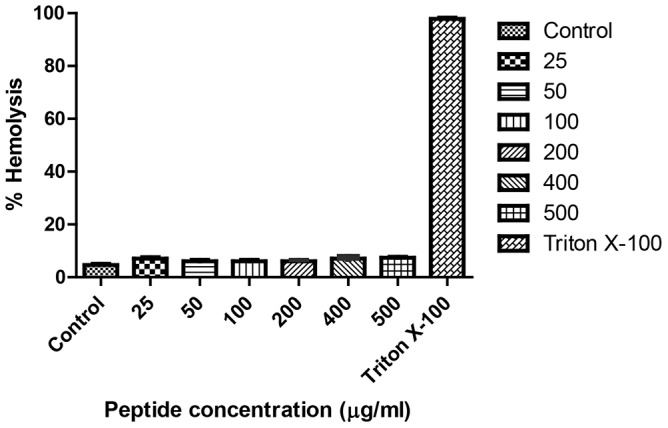
Hemolytic activity of EC5. 1% suspension of chicken RBCs were made with PBS. 100 µl and 50 µl of this suspension were incubated with different concentrations of the peptide in 96 well microtiter plates. Results were read visually.

AMPs that kill bacteria may also exhibit hemolytic activity. The hemolytic activity of EC5 against chRBCs was determined as a measure of peptide toxicity toward higher eukaryotic cells [37], [38]. MHC is the maximal peptide concentration that produces no hemolysis after 24 h of incubation at 37°C. EC5 showed non-hemolytic activity against chRBCs at concentrations up to 500 µg/ml (Fig. 6).

### Cytotoxic activity

**Figure 7:**
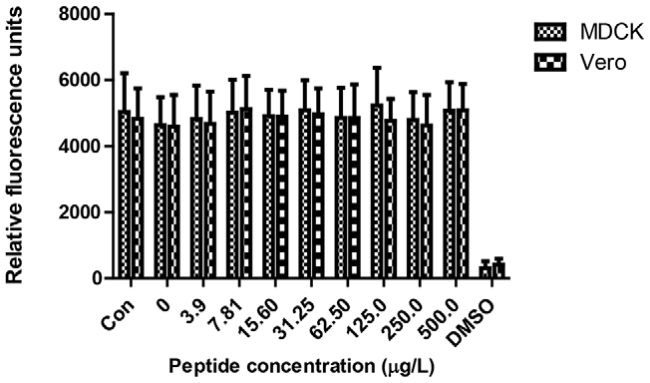
Cytotoxicity of the peptides. MDCK and Vero cells were used to evaluate the toxicity of the peptide EC5 to mammalian cells.

AMPs have gained attention over the recent years. However issues such as cytotoxicity have limited their use. The toxicity of the peptide to MDCK cells and Vero cells was tested by PrestoBlue™ Cell Viability assay (Fig. 7). All tested concentrations of EC5 up to 500 µg/ml showed no cytotoxicity against MDCK and Vero cells.

### Mechanism of action of EC5 Outer membrane depolarization

**Figure 8:**
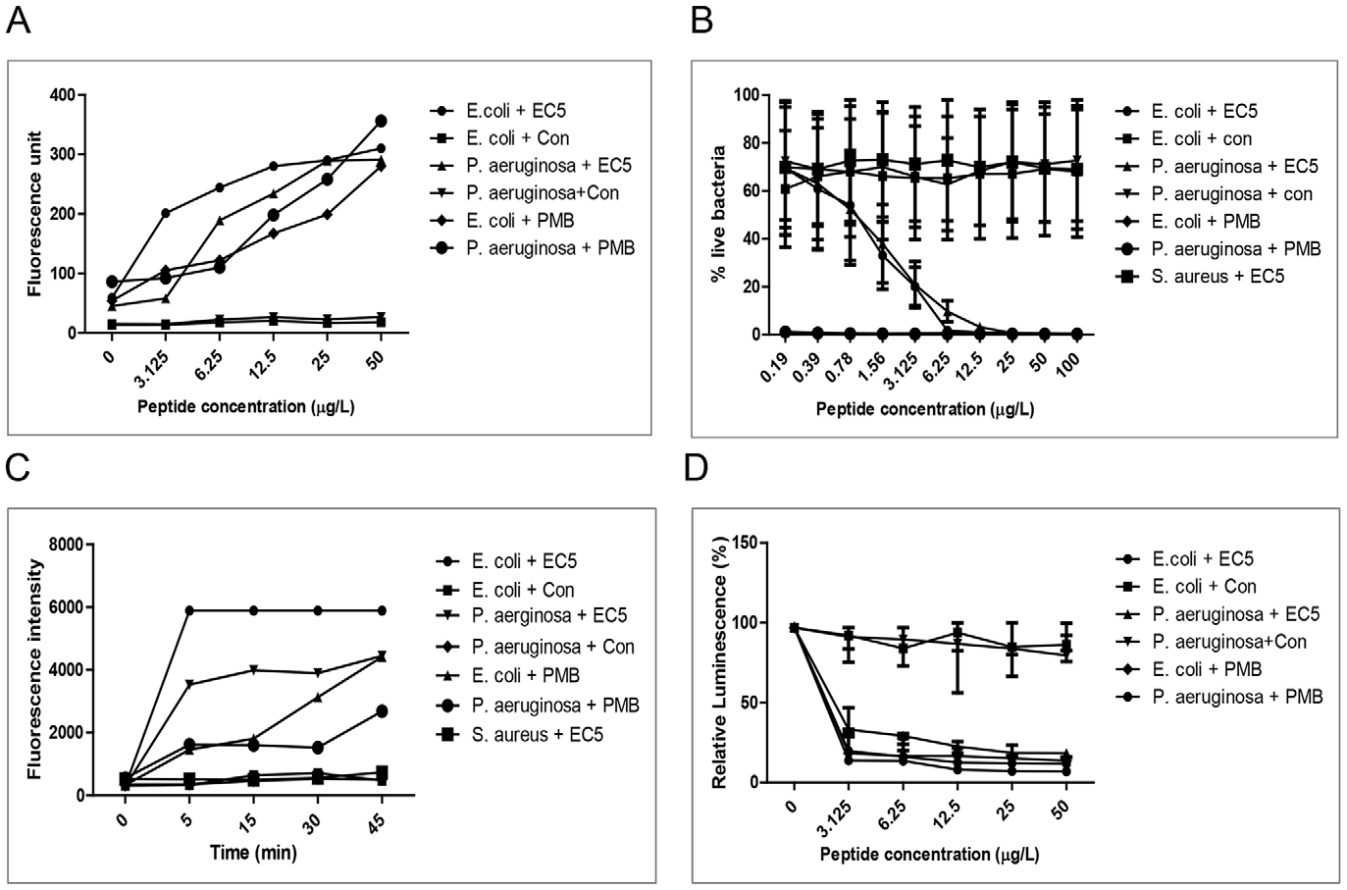
Mechanism of action of EC5 against *E. coli* and *P. aeruginosa*. A. Outer-membrane permeabilization mediated by EC5 as assessed by NPN uptake. Effect of EC5 and Polymyxin B on NPN fluorescence. Value on y axis is the maximum fluorescence upon NPN uptake by the bacteria. B. EC5-induced permeability of bacterial cells studied using Syto9 and PI staining. Peptide treated cells had increased membrane permeability as seen by increase in red fluorescence whereas live or untreated cells showed increase in green fluorescence. C. Cytoplasmic membrane depolarization using the fluorescent dye diSC_3_-5. Corresponding values on y axis represents maximum intensity upon release of the dye mediated by EC5 plotted against time (min). D. EC5 mediated inhibition of ATP synthesis. ATP concentration was measured after the addition of EC5 and polymyxin B at various concentrations and the luminescence units measured.

Outer membrane permeabilizing activity of EC5 against *E. coli* and *P. aeruginosa* was determined using the fluorescent dye N-phenyl-1-napthylamine (NPN) assay. The outer membrane of a bacterial cell is impermeable to NPN under normal conditions. However permeabilization of outer membrane by antimicrobial peptides allows the uptake of NPN thereby leading to increase in fluorescence in the cell. Fig. 8A shows dose-dependent increase in fluorescence in the presence of NPN in *E. coli* and *P. aeruginosa*, indicating that the peptide EC5 was able to disrupt the outer membrane of *E. coli* and *P. aeruginosa*. The outer membrane permeabilizing activity of EC5 was compared to polymyxin B, a well-studied membrane permeabilizing agent [39]. When both *E. coli* and *P. aeruginosa* were incubated with EC5, an increase in fluorescence was observed that was higher than for polymyxin B at similar concentrations.

### Cytoplasmic membrane permeabilization assay

The membrane permeabilizing activity of EC5 was also studied using SYTO9 and PI staining method. SYTO9 stain labels both live and dead bacteria when alone, but propidium iodide stains only cells with damaged membranes, reducing the level of green fluorescent SYTO9 when both the dyes are present. Hence the live cells appear green and the membrane damaged or dead cells appear red. The ratio of green versus red fluorescence can be calculated as a measure of live bacteria. Fig. 8B shows that EC5 exhibited concentration-dependent activity against *E. coli* and *P. aeruginosa*. Polymyxin B, an AMP with a net charge of +5 with membrane permeabilizing activity was used as positive control. Comparison of PI and SYT09 fluorescence showed that polymyxin B was able to permeabilize almost all bacterial cell membranes even at the lowest concentration used. EC5 at 6.25 µg/ml and 12.5 µg/ml showed membrane permeability of *E. coli* and *P. aeruginosa* cells respectively.

### Membrane depolarization

Cytoplasmic depolarization assay(Δψ) was performed using the membrane potential sensitive dye 3,3′–dipropylthiadicarbocyanine iodide (diSC_3_ -5) [7]. This dye can cross the outer membrane of the bacterial cell and become concentrated in the cytoplasmic membrane and thereby it self-quenches its own fluorescence. Upon addition of a membrane-permeabilizing agent the dye is released with consequent increase in the fluorescence. EC5 at 8 µg/ml and 16 µg/ml caused rapid depolarization of the cytoplasmic membrane in both *E. coli* and *P. aeruginosa* resulting in the release of diSC_3_-5 within 5 min (Fig. 8C). The killing was also rapid, resulting in complete inhibition of CFU within 5 min of exposure of the peptide. However Polymyxin B even at its MIC of 0.5 µg/ml caused slow release of diSC_3_ -5 when compared to EC5. *S. aureus* with EC5 showed no trace of release of diSC_3_ -5 even after 45 min at 64 µg/ml.

### ATP inhibition by EC5

ATP inhibition as a measurement of microbial viability was measured using BacTiter-Glo™ kit (Invitrogen, USA). Intracellular ATP levels of *E. coli* and *P. aeruginosa* were examined after the addition of different concentrations of EC5 (Fig. 8D). ATP inhibition began 5 min after the addition of EC5 at lowest concentration of 3.125 µg/ml which was slightly lower than the MIC of 8 µg/ml for *E.coli* and *P. aeruginosa*. The level of ATP did not rise after the addition of EC5. These results suggest that EC5 disrupts the cytoplasmic membrane thereby resulting in reduced ATP levels, leading to cell death.

### Molecular Dynamics

**Figure 9:**
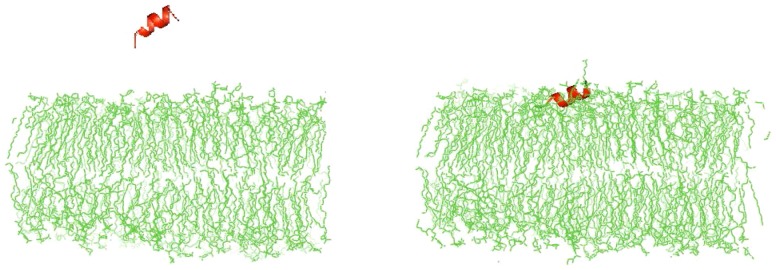
AMP- bacterial membrane interaction studied by molecular dynamic simulations. EC5 was simulated with a POPE∶POPG membrane bilayer model using the Cluspro 2.0 and Hex protein docking server. PDB files generated were visualized using PYMOLv0.99.

*In-silico* studies of EC-5 lipid interactions were performed by Cluspro 2.0 and Hex protein docking server. EC5 was docked with POPE∶POPG lipid bilayer. 3D model structures were visualized using PYMOLv0.99. Docking studies showed that EC5 was adsorbed onto the surface of the lipid bilayers by adopting an orientation parallel to the lipid surface and thereby causing a disruption of the lipid headgroup packing (Fig. 9). EC5 also showed penetration into the lipid bilayers suggesting the degree of peptide interaction with POPE∶POPG.

## Discussion

Emergence of antibiotic resistant strains due to widespread use of antibiotics and dearth of new antibiotics has resulted in looking for new antimicrobial agents with new targets and unique mechanism of action. AMPs are gaining importance due to their superior and dynamic mechanism of action compared to antibiotics [40]. AMPs have existed for millions of years; however resistance to antimicrobial peptides has not been reported. Computer-assisted peptide design, combinatorial libraries and structure based designs are some of the methods used for designing novel AMPs [41]. Another way to develop novel antimicrobial peptides is by using recombinant bacteriophages engineered to display short random peptide coding sequences in their genome. Combinatorial phage-display is a powerful tool for the selection of short peptides binding to any target, biological or non-biological [42], [43]. Cell surface recognition is considered to be a crucial event in biological events. Finding new scaffolds that recognize cell surface can be useful in diagnostics and therapeutics [44]. Since cell surfaces are composed of complex molecular compositions they can provide unique binding sites. One approach to identify ligands that binds to cell surfaces is by using phage-display. Phage-display has been used successfully in number of applications, including vaccine development, protein drug discovery, and to generate diagnostic and therapeutic peptides [19]–[21], [23], [43], [45]–[48]. Several peptides with antimicrobial property have been selected by phage-display indicating that phage-display libraries play an important role in drug development [12], [14], [19], [22], [45]–[47]. Phage-display peptide as inhibitors against essential bacterial enzymes is another approach that scientists are using to develop novel antimicrobial peptides. A recent study used phage-display to select peptides against the enzyme I component of the *E. coli* phosphotransferase system (PTS) thereby inhibiting the cell growth [12], [14]. This enzyme is present only in bacteria and is absent in eukaryotes thereby making it highly selective and specific. In this study we used a whole-cell phage-display approach to identify peptides binding to the cell surface of *E. coli*. By using this approach we observed that a specific sequence, represented by EC5 (RLLFRKIRRLKR) repeated multiple times (5 out of 10 clones). Interestingly the aligned sequences contained conserved Arginine (R) and Lysine (L) residues. These Arginine and Lysine residues have been shown to be major components of antimicrobial peptides [49]. Majority of native antimicrobial peptides have net charge ranging from +2 to +8 and hydrophobic value ranging from 41% to 50% [50]–[52]. EC5 showed features common to antimicrobial peptides: net positive charge of +7 and hydrophobic value of 41%. Sequence analysis of EC5 suggested that it was a cationic α-helical peptide.

EC5 showed antimicrobial properties deemed bactericidal by structure analysis of the peptide and hence we investigated the bactericidal activity of the peptide *in vitro*. The best bactericidal AMP kills bacteria *in vitro*, including certain antibiotic-resistant pathogens, with MICs ranging from 1 to 8 µg/ml [40]. EC5 is a narrow spectrum antibacterial agent and was most effective against gram-negative bacteria tested, with an MIC and MBC of 8 µg/ml for *E. coli* strain. *P. aeruginosa* on the other hand demonstrated an MIC of 8 µg/ml for ATCC strain 27853 and 16–32 µg/ml for *P. aeruginosa* ATCC 12121. The EC5 peptide showed no activity against Gram-positive strains and appeared to be more active against Gram-negative strains with a MIC of 4–128 µg/ml. Since AMPs are known to often show reduced MIC values when full MHB medium is used for testing, we also tested the MIC values of EC5 using cation-supplemented MHB. However we found no difference in the MIC values of EC5 when tested by both the methods suggesting that the presence of salts in MHB does not interfere with the activity of EC5.

While AMPs are effective *in vitro*, they may lose their activity *in vivo*, when given intravenous since human blood may have factors such as proteins or small nucleic acids that can adsorb AMPs and hinder their activity. In this study we developed an ex vivo assay using human plasma and platelets as the test medium to evaluate the extent and duration of EC5 efficacy. The EC5 peptide was incubated along with the test organisms into the medium and incubated for 2 h. In this experimental setup, EC5 exhibited potent bactericidal activity in homologous plasma and inhibited *E. coli* and *P. aeruginosa* at concentration of 50 µg/ml. However, at lower concentration it did not retain the similar effect as in conventional media. EC5 in the presence of platelets suspended in plasma showed complete inhibition of *E. coli* at 25 and 50 µg/ml. However at 12.5 µg/ml it showed only 4.5 log_10_ reduction in CFU/ml; Also, the activity of EC5 was lower against *P. aeruginosa* in the presence of platelets suspended in plasma compared to what is observed in conventional media. These observations suggest that some plasma factors may be interfering with, or masking the effect of EC5 at lower concentrations.

Safety analysis such as the non-hemolysis of chRBCs (even at concentration of 500 µg/ml) and non-cytotoxicity of the peptide suggests a potential for EC5 to be an effective drug candidate. Since the peptide MIC and peptide concentrations inducing hemolysis differ by more than an order of magnitude, the data indicates that EC5 therapeutic index for the treatment of bacterial infections could be favorable. Many AMPs kill bacterial cells by disrupting their membrane integrity. We investigated the interaction of peptides with membranes using membrane permeabilization assays. EC5 caused rapid increase in outer membrane permeabilization at lower concentration, below MBC, which was followed by cytoplasmic depolarization. The changes correlated well with cell killing and cytoplasmic depolarization at the same time. However, polymyxin B at 3.125 µg/ml resulted in complete inhibition of CFU within 5 min of exposure of the peptide, but only minimal release of diSC_3_5 from the cells after 5 min of exposure. Polymyxin B causes cell death prior to cytoplasmic depolarization whereas for EC5 both the events appear to occur at the same time. Our observations suggest that EC5 may disrupt the cytoplasmic membrane, causing rapid depolarization, and inhibition of macromolecular synthesis as seen by ATP inhibition and rapid cell death. In order to confirm our hypothesis we also investigated the peptide-membrane interaction using molecular dynamic simulations. EC5 was simulated with POPE∶POPG membrane bilayer model using the Hex docking server (http://hexserver.loria.fr/) and Cluspro protein-protein docking server (Version 2.0). Docking results suggested that EC5 may lie parallel to the membrane and translocate across the cytoplasmic membrane. These results suggest that EC5 penetrates bacterial-mimicking membranes as a result of electrostatic interactions which are essential for peptides to interact with membrane surface. The peptide then integrates into the cell membrane, causing depolarization and cell death [53]. Currently Gram-negative bacteria such as *E. coli* and *P. aeruginosa* are causing concern due to the rapid spread of extremely resistant strains to traditional antibiotics.

The use of polymyxin B was abandoned previously since the antibiotic showed high toxicity, especially nephrotoxicity [54]–[56]. EC5 showed potent *in vitro* and low cytotoxicity, suggesting their use as drug template for the development of new antibacterial drugs. Since EC5 shows membrane-permeabilizing properties it can also be used in combination with conventional antibiotics to facilitate the entry of drugs into the cells. Combination therapy with antibiotics can potentially be used to broaden the antimicrobial spectrum to treat multiple-drug resistant strains. [57]. Our future studies would include developing improved analogs of EC5 with improved antimicrobial activity against other pathogens using different approaches such as substitution of amino acids, inclusion of D-amino acids or beta-amino acids and cyclization of peptides and peptoid mimics to name a few. Drug-resistant strains of *E. coli* are a serious health problem and hence AMPs such as EC5 may have potential therapeutic applications. In conclusion, these studies demonstrate that peptides with antimicrobial activity can be selected from random phage libraries and may prove useful in the development of novel bactericidal agents.
